# Hypermagnesaemia, but Not Hypomagnesaemia, Is a Predictor of Inpatient Mortality in Critically Ill Children with Sepsis

**DOI:** 10.1155/2022/3893653

**Published:** 2022-01-27

**Authors:** Huabin Wang, Junbin Huang, Xinghan Jin, Chunmei Chen, Airun Zhang, Yuhui Wu, Chun Chen

**Affiliations:** ^1^Division of Hematology/Oncology, Department of Pediatrics, The Seventh Affiliated Hospital of Sun Yat-Sen University, Shenzhen 518107, China; ^2^Department of Pediatric Intensive Care Unit, The Seventh Affiliated Hospital of Sun Yat-Sen University, Shenzhen 518107, China; ^3^Center of Digestive Disease, The Seventh Affiliated Hospital of Sun Yat-Sen University, Shenzhen 518107, China; ^4^Department of Neonatal Intensive Care Unit, The Seventh Affiliated Hospital of Sun Yat-Sen University, Shenzhen 518107, China; ^5^Department of Pediatric Intensive Care Unit, Shenzhen Children's Hospital, Shenzhen 518000, China

## Abstract

**Objective:**

The effect of serum magnesium on the prognosis of children with sepsis in the pediatric intensive care unit (PICU) is unclear. This study was designed to assess the risk of inpatient mortality for children with sepsis in the PICU based on serum magnesium levels at admission.

**Methods:**

We collected patients' clinical information from the Pediatric Intensive Care database and then performed locally weighted scatterplot smoothing (LOWESS) analysis, Kaplan–Meier analysis, and multivariate logistic regression to determine the relationship between admission serum magnesium and inpatient mortality in children with sepsis.

**Results:**

A total of 974 critically ill children with sepsis were included, with 246 patients in the hypomagnesemia group, 666 in the normal group, and 62 in the hypermagnesemia group. The chi-square test suggested that the hypermagnesemia group had higher in-hospital mortality than the normal group (14.5% vs. 2.4%, *P* < 0.001). Kaplan–Meier curves revealed that the 30-day overall survival rate was lower in the hypermagnesaemia group than in the normal group (*P* < 0.001). The multivariate logistic regression model revealed that hypermagnesaemia was a risk factor related to inpatient mortality (odds ratio 4.22, 95% CI 1.55-11.50), while hypomagnesaemia was not a significant factor for inpatient mortality (odds ratio 0.78, 95% CI 0.26-2.32).

**Conclusion:**

Hypermagnesaemia, but not hypomagnesaemia, is a predictor of inpatient mortality in critically ill children with sepsis.

## 1. Introduction

Sepsis is caused by an imbalanced host response to infection and can lead to systemic multiorgan dysfunction. Sepsis is a serious threat to the lives and health of children. Among the 7.6 million children who die at ages younger than 5 years, 64% die of sepsis or septic shock caused by severe infection [1, 2]. The Surviving Sepsis Campaign launched in 2018 clearly proposed the concept of the “hour-1 bundle,” which emphasized that sepsis should be regarded as an emergency medical event rather than a single disease. Timely intervention is of vital importance, and the identification of risk factors for sepsis on admission can be helpful for patient triage, individualized treatment, and medical decision making [3, 4].

Serum magnesium testing is part of the routine comprehensive biochemistry panel. In recent years, serum magnesium has attracted increasing attention from the medical community as a novel prognostic indicator. Several studies have shown that serum magnesium is related to an increased risk of acute respiratory failure, acute kidney injury, and shock [5–7]. Metabolic disorders involving serum magnesium are common in inpatients [8–11]. Abnormalities in magnesium may affect the prognosis of some diseases. However, to our knowledge, no studies have investigated the relationship between serum magnesium and the prognosis of pediatric intensive care unit (PICU) patients with sepsis, and clinicians do not have simple indicators to assess the prognosis of children with sepsis. Thus, the aim of this study was to investigate the value of serum magnesium for predicting mortality in children with sepsis.

## 2. Methods

### 2.1. Introduction to the Database

The Pediatric Intensive Care (PIC) database is a large, pediatric-specific, single-center, Chinese-English bilingual database containing the clinical data of 12,881 children admitted to the PICU of Children's Hospital of Zhejiang University School of Medicine between 2010 and 2018 [12]. This project was approved by the Institutional Review Board of this hospital. The need for informed consent was waived because the project was a retrospective study, and clinical decision-making was not affected. All protected health information was deidentified.

### 2.2. Subject Selection

Inclusion criteria: (1) 1 month to 18 years of age; (2) in cases of repeat admissions, only information from the initial admission was used; and (3) children with sepsis: children with suspected or confirmed infections within 48 hours of PICU admission presenting with systemic inflammatory response syndrome.

**Figure 1:**
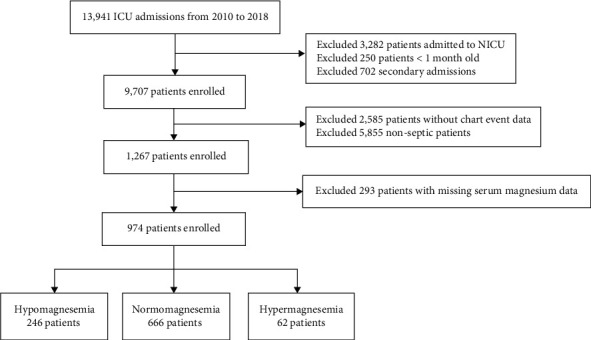
Flow diagram of patient recruitment.

Exclusion criteria: (1) patients admitted to the neonatal intensive care unit; (2) a lack of chart event data; or (3) no data on serum magnesium. The inclusion/exclusion flow chart is shown in Figure 1.

### 2.3. Variable Information and Grouping

The extracted variable information included patient age, sex, laboratory tests (including activated partial thromboplastin time, serum ionized calcium, serum potassium, serum sodium, arterial lactic acid, arterial pH, white blood cell count, and platelet count), and complications (including acute kidney injury, anemia, congenital heart disease, diabetic ketoacidosis, liver dysfunction, and cancer). All laboratory results were initial values taken after PICU admission. Complications were diagnosed within 48 hours of PICU admission (see Supplementary Table 1 for diagnostic criteria). In this study, missing values, which accounted for less than 5% of all variables, were replaced with the median or mean values. The primary outcome measure was inpatient mortality, which was defined as all-cause death during hospitalization.

Based on the serum magnesium level, the children with sepsis were divided into the hypomagnesaemia group (<0.75 mmol/L), the normal group (0.75 mmol/L ≤ serum magnesium ≤ 1.0 mmol/L), and the hypermagnesaemia group (>1.0 mmol/L).

### 2.4. Statistical Analysis

The mean and standard deviation are used to describe normally distributed data. For skewed data, we employ the median and interquartile range. Nominal data are described as the number in each category and the corresponding percentage [13]. Normally distributed data were compared by the *t*-tests, and skewed data were compared by the Mann–Whitney *U*-test. Nominal data were compared by the chi-square test. The relationship between serum magnesium and 30-day mortality was estimated with locally weighted scatterplot smoothing (LOEWSS). The Kaplan–Meier method was then used to plot the survival curves of children with different serum magnesium levels, which were then analysed with the log-rank test. Multivariate logistic regression models were performed to analyse risk factors for in-hospital mortality, which were corrected with the following extended models: model 1 = age + sex; model 2 = model 1 + (laboratory data); and model 3 = model 2 + (complications). The laboratory data and complications included are described in Section 2.3. variable information and grouping. Factors included in the final model were determined using backward conditional regression. All tests were two-sided, and *P* < 0.05 was considered statistically significant. STATA v.16, SPSS v.24, EmpowerStats software (http://www.empowerstats.com version R 3.6.1) and R package were used for the statistical analysis.

## 3. Results

### 3.1. Baseline Characteristics

A total of 974 critically ill children with sepsis were included, including 509 boys (52.3%) and 465 girls (47.7%) (Table 1). The median age was 32.3 months at PICU admission. The normal group was compared with the hypomagnesaemia group and the hypermagnesaemia group separately. The rates of acute kidney injury and liver dysfunction were higher in the hypomagnesaemia group and the hypermagnesaemia group than in the normal group. No significant among-group differences in the length of hospital stay were noted. The inpatient mortality rate was 6.0 times higher in the hypermagnesaemia group than in the normal group (14.5% vs. 2.4%, *P* < 0.001). However, no significant difference was observed in inpatient mortality between the hypomagnesaemia group and the normal group (2.0% vs. 2.4%, *P* = 0.741).

### 3.2. Preliminary Relationship between Serum Magnesium and Mortality

**Figure 2:**
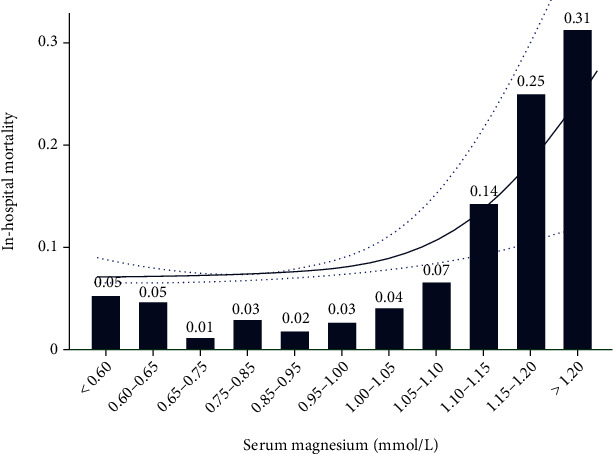
Relationship between serum magnesium and inpatient mortality in critically ill children with sepsis using locally weighted scatterplot smoothing analysis. The solid line shows the curve between serum magnesium and inpatient mortality, and the dashed lines refer to 95% CIs.

LOWESS showed that the mortality rate started to increase significantly once the serum magnesium level reached 1.00 mmol/L (Figure 2). The inpatient mortality rate was 2.0% to 3.0% when the serum magnesium level was 0.75-1.00 mmol/L. However, the inpatient mortality rate was as high as 31.0% when the serum magnesium level was >1.20 mmol/L.

**Figure 3:**
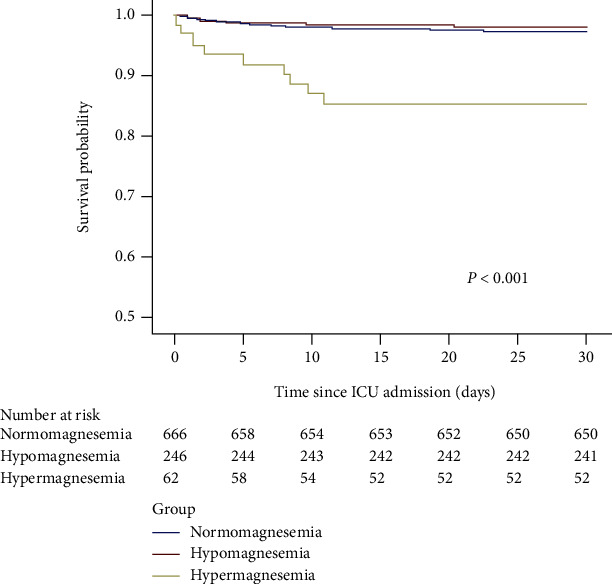
Association between serum magnesium and 30-day overall survival in critically ill children with sepsis.

Kaplan–Meier curves were used to analyse the relationship between the serum magnesium level and 30-day mortality (Figure 3). The overall survival rate was lower in the hypermagnesaemia group than in the normal group (*P* < 0.001), while no difference in the overall survival rate was observed between the hypomagnesaemia group and the normal group.

### 3.3. Further Relationship between Serum Magnesium and Mortality

Table 2 presents the results when serum magnesium (mg/dL) was included as a continuous variable in the multivariate logistic regression model. Both univariate analysis and adjusted multivariate analysis demonstrated a significant odds ratio (OR) for inpatient mortality. In model 3, each 1 mg/dL increase in serum magnesium was associated with a 146% increase in the inpatient mortality rate (OR 2.46, 95% CI 1.29-4.70).

In the backward stepwise multiple logistic regression model, the normal group was used as a reference. Hypermagnesaemia was significantly correlated with an increased inpatient mortality rate (Table 3), as shown in the univariate analysis (OR 6.90, 95% CI 2.91-16.36), model 1 (OR 6.90, 95% CI 2.91-16.36), model 2 (OR 3.15, 95% CI 1.20-8.25), and model 3 (OR 4.22, 95% CI 1.55-11.50). The OR values of other covariates in model 3 are shown in Supplementary Table 2. However, no significant correlation was observed between hypomagnesaemia and mortality, even when the hypomagnesaemia group was further divided into a severe hypomagnesaemia subgroup (<0.70 mmol/L) and a mild hypomagnesaemia subgroup (0.70-0.75 mmol/L), based on the median value (see Supplementary Table 3).

## 4. Discussion

The main purpose of this study was to evaluate whether children with sepsis in the PICU have worse outcomes if they also have magnesium metabolism disorders. The results showed that children with sepsis had a significantly higher risk of inpatient mortality when serum magnesium levels reached 1.0 mmol/L. After confounding factors such as acute kidney injury and calcium and potassium metabolic disorders were controlled, hypermagnesaemia was the only factor associated with increased mortality.

Magnesium, an important mineral, is the third most common intracellular ion after potassium and calcium. Magnesium plays an important role in maintaining the normal physiological functions of the body because it is a cofactor in hundreds of enzyme systems. Its functions include the regulation of nucleic acid and protein synthesis, neuromuscular conduction, signal transduction, blood sugar control, and blood pressure regulation [14]. Children with sepsis have serious and complex systemic inflammatory responses and are prone to electrolyte disturbances, including magnesium disturbance. Previous studies have shown that 40% to 60% of PICU patients have hypomagnesaemia [8, 11]. Hypermagnesaemia is considered relatively uncommon [8]. This study showed that patients with hypomagnesaemia outnumber those with hypermagnesaemia by 4.0-fold, and the distribution pattern of magnesium changes is consistent with the findings of previous studies.

This study showed that hypermagnesaemia was significantly correlated with increased inpatient mortality in children with sepsis. A study analyzing the relationship between admission serum magnesium and inpatient mortality in 9,005 patients with acute myocardial infarction found that both high-normal magnesium levels (2.2-2.4 mg/dL) and hypermagnesaemia (>2.4 mg/dL) were independent risk factors for inpatient mortality in patients with acute myocardial infarction (adjusted OR 1.63 vs. 1.39) [15]. In a retrospective cohort study, Haider et al. included 5339 patients treated at the emergency department [16]. Of these, 352 had hypomagnesaemia, with a mortality rate of 6.3%, and 151 had hypermagnesaemia, with a mortality rate of 36.9%. In a multivariate Cox regression model, hypermagnesaemia was a powerful independent risk factor for inpatient mortality (hazard risk (HR) 11.6, 95% CI 7.3-18.5), while hypomagnesaemia was unrelated to mortality (HR 1.7, 95% CI 0.9-3.0). In a study of 8498 cardiac ICU patients, each 1-unit (mg/dL) increase in serum magnesium was correlated with a 74% increase in inpatient mortality (OR 1.74, 95% CI 1.39-2.18), which is consistent with the findings of this study [17].

Several factors explain why hypermagnesaemia is associated with increased inpatient mortality in children with sepsis. First, magnesium is an effective extracellular and intracellular calcium channel blocker. Meanwhile, intracellular magnesium profoundly blocks a variety of cardiac potassium channels. The combination of these changes further damages cardiac function and causes malignant arrhythmias and heart failure [18]. Second, magnesium exerts its effect on blood pressure via its mediating effect on vessel dilation [19], transient receptor potential M [20], and aldosterone [21]. In a study of more than 10,000 critically ill patients, hypermagnesaemia was independently correlated with lower systolic blood pressure and the need for vasopressors during the first 24 hours of ICU admission [22]. Third, increased magnesium reduces the transmission of impulses through the neuromuscular junction, resulting in a curare-like response and subsequent drowsiness, loss of deep tendon reflexes, and muscle paralysis, which may lead to flaccid tetraplegia, weakened breathing, and ultimately apnoea, especially in patients with hypocalcaemia [23, 24].

Different studies have reached different conclusions regarding the relationship between hypomagnesaemia and patient mortality. Some studies showed that the incidence of hypomagnesaemia was high in critically ill patients, and hypomagnesaemia was correlated with increased mortality in critically ill patients and patients with sepsis [25–28]. In a retrospective study of 100 children in the PICU, the mortality rate was 9.1 times higher in patients with hypomagnesaemia (19/63, 30%) than in patients with normal magnesium levels (1/30, 3.3%) [8]. In 2010, Santos et al. conducted a prospective observational study to evaluate 54 patients with acquired immunodeficiency syndrome who developed acute kidney injury during hospitalization [29]. The results of the logistic regression model suggested that hypomagnesaemia was the only relevant factor for persistent renal dysfunction (OR 6.9, 95% CI 1.2-40.0, *P* = 0.030) and death (OR 6.9, 95% CI 1.2-40.8, *P* = 0.033). However, these studies generally had small sample sizes or did not control for confounding factors.

Unlike previous studies, this study showed that hypomagnesaemia was unrelated to increased mortality, even when patients with hypomagnesaemia were further divided into a severe hypomagnesaemia subgroup and a mild hypomagnesaemia subgroup. Limited clinical and basic investigations of magnesium deficiency have provided evidence that hypomagnesaemia is related to free radicals, cytokines, neuropeptides, endotoxins, endogenous antioxidants, and vascular permeability [30, 31]. However, the actual clinical relevance of these results is unclear, and they may cause abnormal neuromuscular performance and calcium and potassium metabolic disorders [32]. The results of the present study cast doubt on these relationships. First, most of the previous studies had small sample sizes and did not control for important confounding factors such as renal dysfunction and electrolyte disturbances. Second, hypomagnesaemia may be related to increased mortality in specific populations, such as elderly patients, patients with varying degrees of chronic kidney disease, and patients with heart failure [33–35]. In 2015, Cheungpasitporn et al. conducted a retrospective analysis of 28,812 hospitalized patients and found that the survival rate was low for patients with hypomagnesaemia or hypermagnesaemia [9]. After controlling for all variables except the diagnosis at admission, serum magnesium < 1.7 mg/dL was correlated with an increased risk of inpatient mortality. However, when the diagnosis at admission was controlled for, hypomagnesaemia was no longer a predictor of inpatient mortality, suggesting that hypomagnesaemia had disease-specific effects. This finding may be related to the complexity of magnesium biology, including the interactions among different organ systems and the interdependence of magnesium, potassium, and calcium regulation [36]. Third, the use of different magnesium supplementation strategies at different hospitals may contribute to these contradictory results in single-centre studies.

In the present study, we used a large amount of clinical information from the PIC database and controlled for important covariates. This was the first study to demonstrate a relationship between serum magnesium and mortality in children with sepsis. However, this study has some limitations. First, we did not have access to detailed preadmission information, which may have affected serum magnesium levels on admission. Second, due to the limitations of the database, including the lack of information, such as drug use, the purpose of drugs, and the lack of course records, we could not accurately judge whether each patient's presentation was in line with the diagnosis of septic shock; thus, we could not conduct subgroup analysis. Meanwhile, some unavailable covariates may confuse the predictive effect of serum magnesium. Third, magnesium measurement is a test that is easily overlooked by clinicians, who may not consider a review even if an abnormality in magnesium is detected. Therefore, the database lacks sufficient dynamic data to build a trajectory prediction model. We are planning to prospectively and dynamically collect serum magnesium data from patients with sepsis to construct a prediction model for the entire trajectory of magnesium during hospitalization to further illustrate the predictive value of serum magnesium.

## 5. Conclusion

Hypermagnesaemia is an independent predictor of inpatient mortality in critically ill children with sepsis, suggesting that serum magnesium may be a useful prognostic biomarker for children with sepsis. Serum magnesium should be tested at PICU admission, especially given the adverse outcomes associated with hypermagnesaemia and the low cost of magnesium testing. The results of this study serve as a reminder that healthcare professionals should be careful when giving magnesium to patients without hypomagnesaemia. Further studies, especially randomized controlled trials, are needed to evaluate whether reducing serum magnesium levels can improve the outcomes of children with sepsis.

## Figures and Tables

**Table 1:** Baseline characteristics.

Variable	Hypomagnesemia (*n* = 246)	Normomagnesemia (*n* = 666)	Hypermagnesemia (*n* = 62)	*P* value 1 ^∗^	*P* value 2 ^∗∗^
Age, months	47 (22, 90)	28.3 (14.6, 66.9)	18.7 (12.1, 45.6)	<0.001	0.027
Male, *n* (%)	121 (49.2)	353 (53.0)	35 (56.5)	0.306	0.603
Laboratory data					
APTT, s	29.7 (26.6, 32.9)	30.0 (26.9, 34.6)	31.5 (25.9, 38.0)	0.124	0.547
Ionized calcium, mmol/L	1.21 (1.13, 1.25)	1.22 (1.14, 1.28)	1.22 (1.18, 1.32)	0.010	0.228
Potassium, mmol/L	3.6 (3.3, 4.0)	3.7 (3.3, 4.0)	3.9 (3.5, 4.3)	0.037	0.010
Lactate, mmol/L	2 (1.3, 3.1)	1.8 (1.3, 2.8)	2.2 (1.4, 3.6)	0.244	0.061
Sodium, mmol/L	137 (135, 139)	137 (135, 140)	138 (135, 141)	0.059	0.305
PH	7.36 (7.31, 7.40)	7.37 (7.33, 7.42)	7.35 (7.25, 7.40)	0.005	0.015
Platelet, 10^9^/L	271 (186, 387)	245 (159, 348)	263 (169, 397)	0.003	0.213
White blood cell, 10^9^/L	12.7 (7.8, 17.6)	11.0 (6.7, 16.1)	12.0 (5.7, 16.5)	0.053	0.730
Comorbidities, *n* (%)					
Acute kidney injury	25 (10.2)	112 (16.8)	20 (32.3)	0.013	0.003
Anemia	188 (76.4)	517 (77.6)	45 (72.6)	0.700	0.365
Congenital heart disease	37 (15.0)	256 (38.4)	12 (19.4)	<0.001	0.003
Diabetic ketoacidosis	22 (8.9)	94 (14.1)	13 (21.0)	0.038	0.145
Liver dysfunction	40 (16.3)	74 (11.1)	16 (25.8)	0.037	0.001
Malignancy	33 (13.4)	15 (2.3)	1 (1.6)	<0.001	0.743
Clinical outcome					
Hospital LOS (day)	12.9 (8.8, 21)	13 (8.9, 19.6)	13.7 (7.6, 23.1)	0.568	0.678
Hospital mortality, *n* (%)	5 (2.0)	16 (2.4)	9 (14.5)	0.741	<0.001

^∗^ *P* value 1 represents the *P* value for the comparison between the group of hypomagnesemia and the group of normomagnesemia. ^∗∗^*P* value 2 represents the *P* value for the comparison between the group of hypermagnesemia and the group of normomagnesemia. APTT: activated partial thromboplastin time; LOS: length of stay.

**Table 2:** Odds ratio (95% confidence interval) for all-cause mortality across serum magnesium levels.

	Serum magnesium as a continuous variable (per 1 mg/dL increase)		
	Odds ratio	95% confidence interval	*P*
Univariate	2.61	1.45 to 4.72	0.001
Model 1	2.78	1.54 to 5.02	0.001
Model 2	2.14	1.15 to 3.97	0.016
Model 3	2.46	1.29 to 4.70	0.006

**Table 3:** Odds ratio (95% CI) for all-cause mortality across three serum magnesium levels.

	Normomagnesemia		Hypomagnesemia		Hypermagnesemia	
	OR (95% CI)	*P*	OR (95% CI)	*P*	OR (95% CI)	*P*
Hospital mortality						
Univariate	Reference	—	0.84 (0.31-2.33)	0.741	6.90 (2.91-16.36)	<0.001
Model 1	Reference	—	0.84 (0.31-2.33)	0.741	6.90 (2.91-16.36)	<0.001
Model 2	Reference	—	0.84 (0.29-2.45)	0.746	3.15 (1.20-8.25)	0.020
Model 3	Reference	—	0.78 (0.26-2.32)	0.654	4.22 (1.55-11.50)	0.005

OR: odds ratio.

## Data Availability

The data presented in this study are available on request from the first author (wanghb53@mail2.sysu.edu.cn). Reanalysis of the data requires approval by the PIC database.

## Supplementary Materials

Supplementary 1 Supplementary Table 1: diagnostic criteria for complications.

Supplementary 2 Supplementary Table 2: logistic regressions of serum magnesium for in-hospital mortality.

Supplementary 3 Supplementary Table 3: a multivariate logistic regression model was used to further clarify the relationship between hypomagnesemia and hospital mortality using a stepwise backward elimination method among septic children.
