# Supplementary material for: Hypermagnesaemia, but Not Hypomagnesaemia, Is a Predictor of Inpatient Mortality in Critically Ill Children with Sepsis
Source: Dis Markers. 2022 Jan 27;2022:3893653. doi: 10.1155/2022/3893653 (PMC8814719; doi:10.1155/2022/3893653)
Supplement: Supplementary 1 — Supplementary Table 1: diagnostic criteria for complications. [file 3893653.f1.docx]

**Supplementary Table 1:** Diagnostic criteria for complications.

| **Complications** | **Diagnostic criteria** |
| --- | --- |
| Acute kidney injury | We used the pROCK criterion, which defines acute kidney injury as an increase in creatinine levels of ≥ 20 µmol/L and ≥ 30% within 7 days. |
| Anemia | 1-4 months < 90 g/L;  4-6 months < 100 g/L;  6-60minths < 110 g/L;  60-144months < 115 g/L;  >144 months < 120 g/L. |
| Congenital heart disease | A scenario of anatomic abnormalities due to dysgenesis or dysplasia of the heart and great vessels, or failure to close (normal in the genus fetus) of a channel that should automatically close after birth. |
| Diabetic ketoacidosis | Blood glucose > 11 mmol/L; venous blood pH < 7.3 or serum bicarbonate < 15 mmol / L; presence of ketone bodies in blood or urine. |
| Liver dysfunction | Total bilirubin > 68.4mmol/L or alanine aminotransferase elevation more than two times the upper value. |
| Malignancy | Including solid malignancies and non-solid malignancies. |
