# Supplementary material for: Hypermagnesaemia, but Not Hypomagnesaemia, Is a Predictor of Inpatient Mortality in Critically Ill Children with Sepsis
Source: Dis Markers. 2022 Jan 27;2022:3893653. doi: 10.1155/2022/3893653 (PMC8814719; doi:10.1155/2022/3893653)
Supplement: Supplementary 2 — Supplementary Table 2: logistic regressions of serum magnesium for in-hospital mortality. [file 3893653.f2.doc]

**Supplementary Table 2: Logistic regressions of serum magnesium for in-hospital mortality.**

|  | Crude OR | 95% CI | *P* | Adjusted OR | | 95% CI | | *P* | |
| --- | --- | --- | --- | --- | --- | --- | --- | --- | --- |
| Age |  | - |  |  |  | |  | |  |
| < 12 months | Ref.  - | | | Ref. | | | | |  |
| ≥12months and < 60 months | 0.84 | 0.35-2.03 | 0.694 | 1.04 | 0.38-2.81 | | 0.944 | |  |
| ≥ 60 months | 0.30 | 0.09-1.03 | 0.055 | 0.25 | 0.07-1.00 | | 0.049 | |  |
| Gender (female) | 0.63 | 0.29-1.33 | 0.221 | 0.71 | 0.30-1.64 | | 0.418 | |  |
| APTT (> 45, s) | 4.09 | 1.76-9.50 | 0.001 | 0.71 | 0.31-1.65 | | 0.425 | |  |
| Calcium (< 1.2 or > 1.3, mmol/L) | 2.10 | 0.93-4.77 | 0.076 | 1.87 | 0.74-4.71 | | 0.187 | |  |
| Potassium (< 3.5 or > 5.5, mmol/L) | 1.53 | 0.74-3.20 | 0.253 | 2.55 | 1.10-5.93 | | 0.029 | |  |
| Lactate (≥2.0, mmol/L ) | 3.08 | 1.40-6.79 | 0.005 | 1.95 | 0.80-4.79 | | 0.143 | |  |
| Sodium (< 135 or > 145, mmol/L) | 3.33 | 1.60-6.93 | 0.001 | 2.22 | 0.99-5.00 | | 0.053 | |  |
| PH (< 7.35 or > 7.45) | 2.18 | 0.99-4.81 | 0.054 | 1.32 | 0.55-3.17 | | 0.542 | |  |
| Platelet (< 100, 10^9^/L) | 3.92 | 1.62-9.47 | 0.002 | 1.04 | 0.33-3.29 | | 0.945 | |  |
| WBC (< 4 or > 12, 10^9^/L) | 2.43 | 1.07-5.52 | 0.034 | 3.43 | 1.35-8.73 | | 0.010 | |  |
| Acute kidney injury | 5.65 | 2.70-11.81 | <0.001 | 4.45 | 1.89-10.45 | | 0.001 | |  |
| Anemia | 0.981 | 0.42-2.32 | 0.965 | 0.86 | 0.31-2.37 | | 0.765 | |  |
| Congenital heart disease | 0.15 | 0.04-0.64 | 0.010 | 0.19 | 0.04-0.89 | | 0.035 | |  |
| Diabetic ketoacidosis | 2.47 | 1.08-5.68 | 0.033 | 1.73 | 0.65-4.59 | | 0.272 | |  |
| Liver dysfunction | 7.21 | 3.43-15.14 | <0.001 | 3.73 | 1.60-8.67 | | 0.002 | |  |
| Malignancy | 0.64 | 0.09-4.83 | 0.668 | 0.43 | 0.05-3.58 | | 0.435 | |  |
| Serum magnesium |  |  |  |  |  | |  | |  |
| 0.75-1.00 mmol/L | Ref. | | | Ref. | | | | |  |
| <0.75 mmol/L | 0.84 | 0.31-2.33 | 0.741 | 0.78 | 0.26-2.32 | | 0.654 | |  |
| >1.0 mmol/L | 6.90 | 2.91-16.36 | <0.001 | 4.22 | 1.55-11.50 | | 0.005 | |  |

APTT, activated partial thromboplastin time; OR, odds ratio; WBC, white blood cell.
