# Supplementary material for: Hypermagnesaemia, but Not Hypomagnesaemia, Is a Predictor of Inpatient Mortality in Critically Ill Children with Sepsis
Source: Dis Markers. 2022 Jan 27;2022:3893653. doi: 10.1155/2022/3893653 (PMC8814719; doi:10.1155/2022/3893653)
Supplement: Supplementary 3 — Supplementary Table 3: a multivariate logistic regression model was used to further clarify the relationship between hypomagnesemia and hospital mortality using a stepwise backward elimination method among septic children. [file 3893653.f3.doc]

**Supplementary Table 3: A multivariate logistic regression model was used to further clarify the relationship between hypomagnesemia and hospital mortality using a stepwise backward elimination method among septic children.**

|  | Adjusted OR | | 95% CI | | *P* | |
| --- | --- | --- | --- | --- | --- | --- |
| Age |  |  | |  | |  |
| < 12 months | Ref. | | | | |  |
| ≥12months and < 60 months | 1.03 | 0.38 to 2.80 | | 0.950 | |  |
| ≥ 60 months | 0.25 | 0.06 to 0.99 | | 0.048 | |  |
| Gender (female) | 0.71 | 0.31 to 1.66 | | 0.430 | |  |
| APTT (> 45, s) | 1.18 | 0.38 to 3.61 | | 0.776 | |  |
| Calcium (< 1.2 or > 1.3, mmol/L) | 1.85 | 0.73 to 4.68 | | 0.192 | |  |
| Potassium (< 3.5 or > 5.5, mmol/L) | 2.56 | 1.10 to 5.94 | | 0.029 | |  |
| Lactate (≥2.0, mmol/L ) | 1.95 | 0.80 to 4.78 | | 0.145 | |  |
| Sodium (< 135 or > 145, mmol/L) | 2.19 | 0.97 to 4.92 | | 0.059 | |  |
| PH (< 7.35 or > 7.45) | 1.34 | 0.56 to 3.24 | | 0.515 | |  |
| Platelet (< 100, 10^9^/L) | 1.03 | 0.33 to 3.26 | | 0.957 | |  |
| WBC (< 4 or > 12, 10^9^/L) | 3.39 | 1.33 to 8.62 | | 0.010 | |  |
| Acute kidney injury | 4.37 | 1.86 to 10.25 | | 0.001 | |  |
| Anemia | 0.84 | 0.31 to 2.34 | | 0.744 | |  |
| Congenital heart disease | 0.19 | 0.04 to 0.88 | | 0.034 | |  |
| Diabetic ketoacidosis | 1.72 | 0.65 to 4.58 | | 0.275 | |  |
| Liver dysfunction | 3.67 | 1.58 to 8.55 | | 0.003 | |  |
| Malignancy | 0.41 | 0.05 to 3.46 | | 0.412 | |  |
| Serum magnesium |  |  | |  | |  |
| 0.75-1.00 mmol/L | Ref. | | | | |  |
| <0.70 mmol/L | 0.88 | 0.27 to 2.91 | | 0.839 | |  |
| 0.70-0.75 mmol/L | 0.54 | 0.07 to 4.35 | | 0.558 | |  |
| >1.0 mmol/L | 4.22 | 1.55 to 11.51 | | 0.005 | |  |

APTT, activated partial thromboplastin time; OR, odds ratio; WBC, white blood cell.
